# Osteoporotic vertebral compression fractures: Increased post-facture mortality, particularly in males

**DOI:** 10.1016/j.bas.2026.107460

**Published:** 2026-09-28

**Authors:** Andreas K. Andresen, Jacob Stampe Frølich, Leah Y. Carreon, Anne Pernille Hermann, Tanja Gram Petersen, Jan Sørensen, Mikkel Ø. Andersen

**Affiliations:** a Center for Spine Surgery and Research, Lillebaelt Hospital, Kolding, Denmark; b Department of Regional Health Research, Faculty of Health Services, University of Southern Denmark, Odense, Denmark; c Department of Medicine, OUH Odense University Hospital, Svendborg, Denmark; d Department of Endocrinology, OUH Odense University Hospital, Odense, Denmark; e OPEN Patient Data Explorative Network, OUH Odense University Hospital & University of Southern Denmark, Odense, Denmark; f School of Population Health, RCSI University of Medicine and Health Sciences, Dublin, Ireland

## Abstract

**Introduction:**

With the on-going demographic shift towards older populations, osteoporosis is an increasing public health issue. Mortality rates after a hip fracture in the elderly have been extensively studied and reported. However, similar information has not been reported after an osteoporotic vertebral compression fracture (OVCF).

**Research question:**

What is the mortality rate after a hospital diagnosed OVCF stratified by sex?

**Materials and methods:**

Using data from the Danish National Health registry, individuals with OVCFs between January 2010 and December 2023 were identified and matched 1:5 with a Control group without OVCF on age, gender, and municipality. Differences in mortality rates at intervals from 30 days to 3 years were calculated and compared for OVCF cases and Controls. A similar analysis was used to explore sex differences for individuals with OVCF.

**Results:**

A total of 37,948 patients with OVCF, 12,693 males and 25,255 females were matched with 63,243 and 123,477 Controls, respectively. Individuals with OVCF had statistically significantly higher mortality rates than Controls. Mortality among individuals with OVCF was statistically significantly higher for males than for females.

**Discussion and conclusions:**

A significantly higher mortality rate among patients with OVCFs compared to non-fracture controls was seen. OVCFs in males were associated with an almost two-fold higher risk of all-cause mortality compared to females. Increased awareness of osteoporosis in the male population and related morbidity should be further addressed in research and clinical practice.

## Highlights

• The study compared 37,948 patients with vertebral fractures to 186,720 controls.
• Mortality rates were described at intervals from 30 days to 3 years.
• Mortality differences were highest during the first months after fracture.
• After 3 years mortality in the fracture group was 26.9% versus 19.5% for controls.
• Adverse impact of a vertebral compression fracture extends beyond the acute phase.

## 1 Introduction

Due to the current demographic shift and the aging global population, osteoporosis is an increasing public health issue (Borgstrom et al., 2020). Osteoporosis is a condition of decreased bone mineral density and deficient skeletal microarchitecture, which causes bones to become weak and prone to low-energy fractures (International Osteoporosis). It is estimated that one in two women and one in five men will have a fragility fracture in their lifetime (Johnell and Kanis, 2004; Kanis et al., 2012). Being considered a disease of women, osteoporosis is often underrecognized in men (Rinonapoli et al., 2021; Fuggle et al., 2024).

The most common fragility fractures include distal radius, hips, and osteoporotic vertebral compression fractures (OVCFs) due to ground level falls (Cummings and Melton, 2002; Hernlund et al., 2013). The high mortality rate after hip fractures in the elderly is widely established (Abrahamsen et al., 2009). Hence the emphasis on early surgical intervention followed by early mobilization (Agarwal et al., 2024) to decrease the incidence of complications associated with decreased mobility such as deep vein thrombosis, pulmonary embolism and general deconditioning. This emphasis on early intervention to prevent complications has not been the case for patients with OVCFs. This may be due to the limited data on the mortality rates after an OVCF.

The objective of this study was to use national registry data to (1) evaluate the incidence of mortality in individuals who have experienced their first osteoporotic vertebral compression fracture in comparison with a matched group of controls, and (2) investigate if there is a difference in mortality for men and women who have experienced their first OVCF.

## 2 Methods

We conducted a registry-based matched cohort study. From the Danish National Patient Registry (Lynge et al., 2011), we identified all patients who were diagnosed in-hospital with a OVCF between 2010 and 2023. We defined individuals with OVCF as patients with a hospital-coded primary or secondary diagnosis of vertebral fracture using the following ICD-10 codes: DS220, DS221, DS320, DS327 and DS328. The date of the first hospital contact with a vertebral fracture was designated as the index date. We restricted the population to individuals over 50 years of age at the time of fracture in accordance with the recommendations by the Danish Health authorities (Authority, 2018). We further exclude patients with a prior or current cancer diagnosis (ICD-10 DC*) to exclude patients with a pathologic fracture. To exclude patients with a traumatic fracture, we excluded all patients with an admission code related to a traumatic injury (BWST1F), and all patients who had a CT-scan performed (UXCE and UXCC) within 2 days of the index fracture.

We linked these individuals to the Danish Civil Registry System (Schmidt et al., 2014) using the national unique personal identification number and obtained data on age, sex and area of residence. Using the civil registry, we identified a control group of five controls matched by age, sex, and area of residence. Level of education was obtained for all cases and controls from the Danish education Registers (Jensen and Rasmussen, 2011). The controls were assigned a similar index date as their matched OVCF case. From the civil registry system, we obtained dates for emigration and death before 31st December 2023 for both groups (Greenland and Morgenstern, 1990; Mansournia et al., 2013).

To account for comorbidities, the Charlson Comorbidity index (CCI)(Charlson et al., 1987, 2022) was constructed based on hospital data from 2000 to 2023 ensuring a ten-year period prior to the index date of the first individuals registered with OVCF. For this analysis we used the following disease categories included in the CCI documentation: diabetes mellitus (ICD-10, E11-14), hypertension (ICD-10, I10-13, 15), ischemic heart disease (ICD-10, I20-25), stroke (ICD-10, I63, 64), congestive heart failure (ICD-10, I50), chronic obstructive pulmonary disease (ICD-10, J44), cancer (ICD-10 codes beginning with “C”), and end-stage renal disease (ICD-10, N18, 19). In addition, information on highest attained educational level was retrieved from the Danish Educational Register and categorized into short, medium, long, and no completed/missing education according to the SEPLINE Guideline (Hjorth et al., 2025).

### 2.1 Statistical analysis

We provide a descriptive analysis of the study population to demonstrate the similarity between the two matched groups. In survival analyses, we followed the study population from the index date until death. Individuals were censored on date of emigration or at the end of the study period, 31-12 2023. Individuals initially selected as controls could subsequently become cases during follow-up. In such situations, follow-up as a control ended at the date of case occurrence, after which the individual entered the study as a case with a new index date. We calculated the cumulative mortality before 30, 90, 180 days and one, two and three years using the Kaplan–Meier method (1 − survival probability) for males and females. We used Cox Regression to estimate hazard ratios (HRs) for one-year mortality among patients with OVCF compared with matched controls, separately for males and females and across age groups. Additionally, we estimated HR for mortality in males versus females with OVCF, where survival time was split in different intervals. The Cox Regression models were adjusted for age and age-squared, indicator variables for the presence of Charlson comorbidity categories, education level, and five regional areas of residence as covariates. We present the estimated HR graphically with 95% confidence intervals. Because some individuals contributed data more than once during follow-up, confidence intervals were estimated accounting for repeated observations within the same individual.

The study was approved by the Hospital Review Board and the Danish Data Protection Agency (jf.nr 22/41225).

## 3 Results

We identified 65,621 individuals who had been diagnosed at a hospital with a vertebral fracture between January 2010 and December 2023. Excluding patients aged 50 and younger, patients with a cancer diagnosis and trauma patients, the study population included 37,948 individuals, 25,255 females and 12,693 males. The matched control group included 186,720 individuals (123,477 females and 63,243 males) (Fig. 1). Females were significantly older than males at the time of fracture, 76.8 years (SD 11.5) versus 71.0 (11.5) (p < 0.001). The age distribution, CCI, educational levels and region of residence are shown in Table 1.

**Fig. 1:**
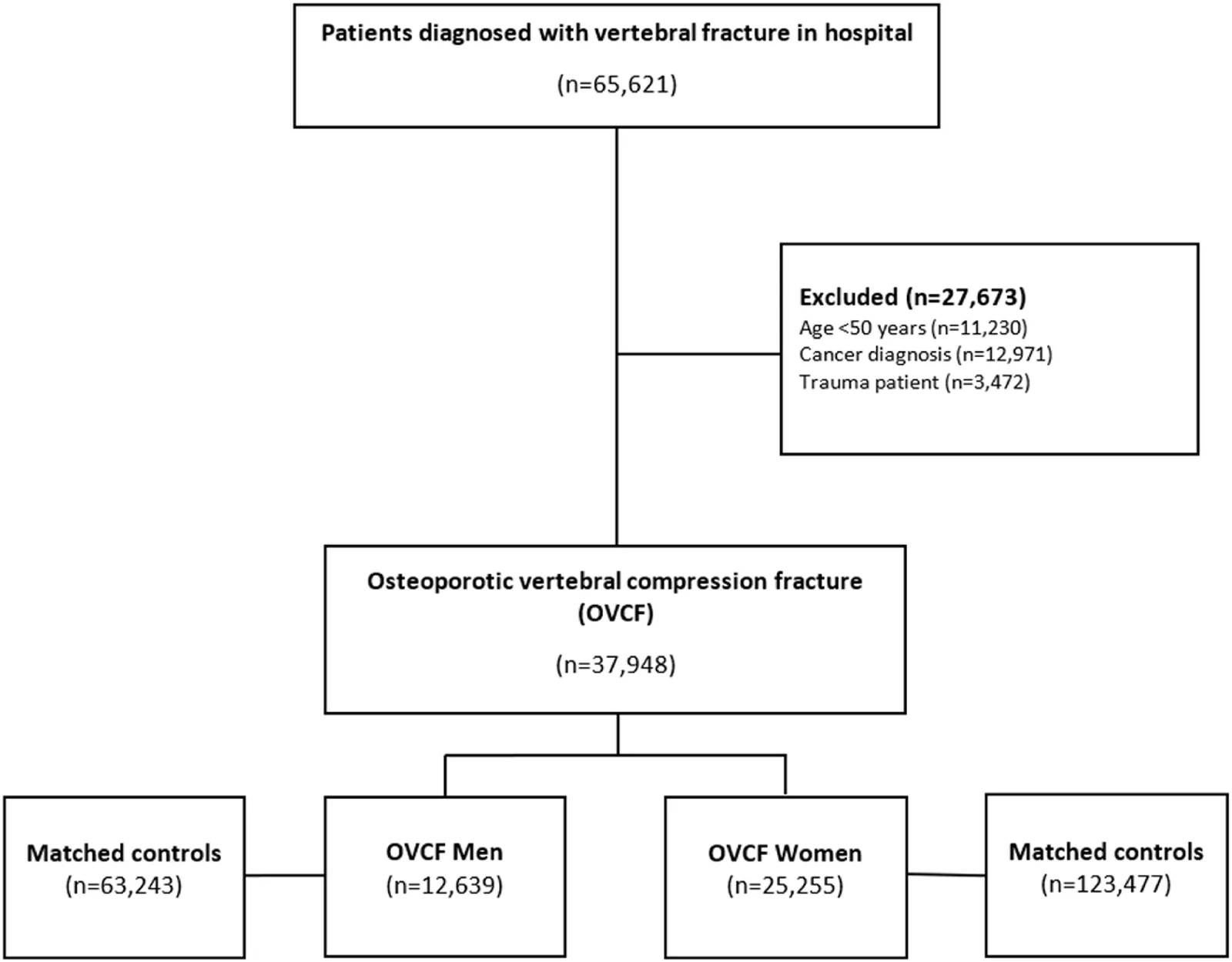
Flowchart of cohort generation from the Danish National Patient Registry.

**Table 1:** Baseline demographics and regional distribution. *Charlson Comorbidity Index. † Highest education level at index.

	Males		Females	
	OVCF	Control	OVCF	Control
N (%)	12,693 (5.6%)	63,243 (28.0%)	25,255 (11.2%)	123,477 (55.2%)
Age groups, n (%)				
50-59	2,587 (20.4%)	13,092 (40.8%)	2,514 (10.0%)	12,815 (10.3%)
60-69	3,181 (25.1%)	16,092 (25.4%)	4,053 (16.0%)	21,421 (17.2%)
70-79	3,511 (27.7%)	17,891 (28.3%)	6,985 (27.7%)	36,560 (29.4%)
80-89	2,731 (21.5%)	13,155 (20.8%)	8,435 (33.4%)	39,366 (31.6%)
90+	683 (5.4%)	3,013 (4.8%)	3,268 (12.9%)	14,315 (11.5%)
No comorbidity (CCI* = 0)	8,526 (67.2%)	50,658 (80.1%)	17,113 (67.8%)	97,213 (76.9%)
CCI = 1	1,715 (13.5%)	5,848 (9.2%)	3,851 (15.2%)	13,292 (10.7%)
CCI ≥ 2	2,452 (19.2%)	6,737 (10.6%)	4,291 (17.0%)	13,972 (11.2%)
Education†, n (%)				
Short education	3,627 (28.6%)	16,832 (26.6%)	10,311 (40.8%)	47,947 (38.5%)
Medium education	5,993 (47.2%)	29,408 (46.5%)	9,196 (36.4%)	45,379 (36.5%)
Long education	2,667 (21.0%)	14,672 (23.2%)	4,566 (18.1%)	24,678 (19.8%)
No completed/missing education	406 (3.2%)	2,331 (3.7%)	1,182 (4.7%)	6,473 (5.2%)
Regional distribution				
North Jutland	1,326 (10.4%)	6,612 (10.5%)	2,371 (9.4%)	11,741 (9.4%)
Central Denmark	2,977 (23.5%)	14,824 (23.4%)	5,686 (22.5%)	28,009 (22.5%)
Southern Denmark	3,274 (25.8%)	16,330 (25.8%)	6,246 (24.7%)	30,831 (24.8%)
Capital Region	3,226 (25.4%)	16,056 (25.4%)	7,017 (27.8%)	34,518 (27.7%)
Region Zealand	1,890 (14.9%)	9,421 (14.9%)	3.935 (15.6%)	19,378 (15.6%)

The difference in cumulative mortality between patients with an OVCF and Controls are shown in Table 2. Mortality was consistently higher in the OVCF group compared to the control group at all time-points. Within 30 days, mortality was 3.9% among males with OVCF compared with 0.7% in male controls, and 3.4% among females with OVCF compared with 0.9% in female controls. We found the largest relative difference in mortality within the first year after sustaining a fracture, however a difference persists over time. At one-year, cumulative mortality reached 15% in males with OVCF versus 7.8% in controls, while corresponding figures for females were 14.2% and 9.5%, respectively. Overall, cumulative mortality in the study population was 26.9% in the OVCF group and 19.5% in the control group at three years. Adjusted analyses showed higher one-year mortality among patients with OVCF compared with Controls across all age groups. Overall, males with OVCF had a 90% higher mortality than matched Controls (HR 1.89 [95% CI 1.79 to 2.00]). HRs were similar for males and females among individuals younger than 70 years, whereas males aged 70 years or older consistently showed higher HR than females (Fig. 2). For the cohort with OVCF, the HR of death 30 days after the first hospital admission for males compared with females was 1.72 [95%CI: 1.53; 1.93], which remained significantly higher three years after OVCF (Fig. 3).

**Table 2:** Cumulative mortality according to sex.

	Males		Females		Total	
	OVCF*	Control	OVCF	Control	OVCF	Control
N (%)	12,693	63,243	25,255	124,477	37,948	187,720
Death before 30 days, n (%)	495 (3.9%)	432 (0.7%)	843 (3.4%)	1,155 (0.9%)	1,388 (3.5%)	1,587 (0.8%)
Death before 90 days, n (%)	922 (7.3%)	1,266 (2.0%)	1,697 (6.8%)	3.144 (2.6%)	2,619 (6.9%)	4.410 (2.3%)
Death before 180 days, n (%)	1,277 (10.2%)	2,418 (3.9%)	2,412 (9.7%)	6.014 (4.9%)	3,689 (9.7%)	8,432 (4.5%)
Death before 1 year, n (%)	1,853 (15.0%)	4,697 (7.8%)	3,485 (14.2%)	11,285 (9.5%)	5,338 (14.1%)	15,982 (8.5%)
Death before 2 years, n (%)	2,738 (22.8%)	8,186 (14.0%)	5,283 (22.0%)	19,577 (17.2%)	8,021 (21.1%)	27.763 (14.8%)
Death before 3 years, n (%)	3,405 (29.2%)	10,849 (19.4%)	6,799 (29.8%)	25,688 (23.6%)	10,204 (26.9%)	36,537 (19.5%)

OVCF*osteoporotic vertebral compression fracture. Percentages represent cumulative mortality estimated using the Kaplan–Meier method (1 − survival probability).

**Fig. 2:**
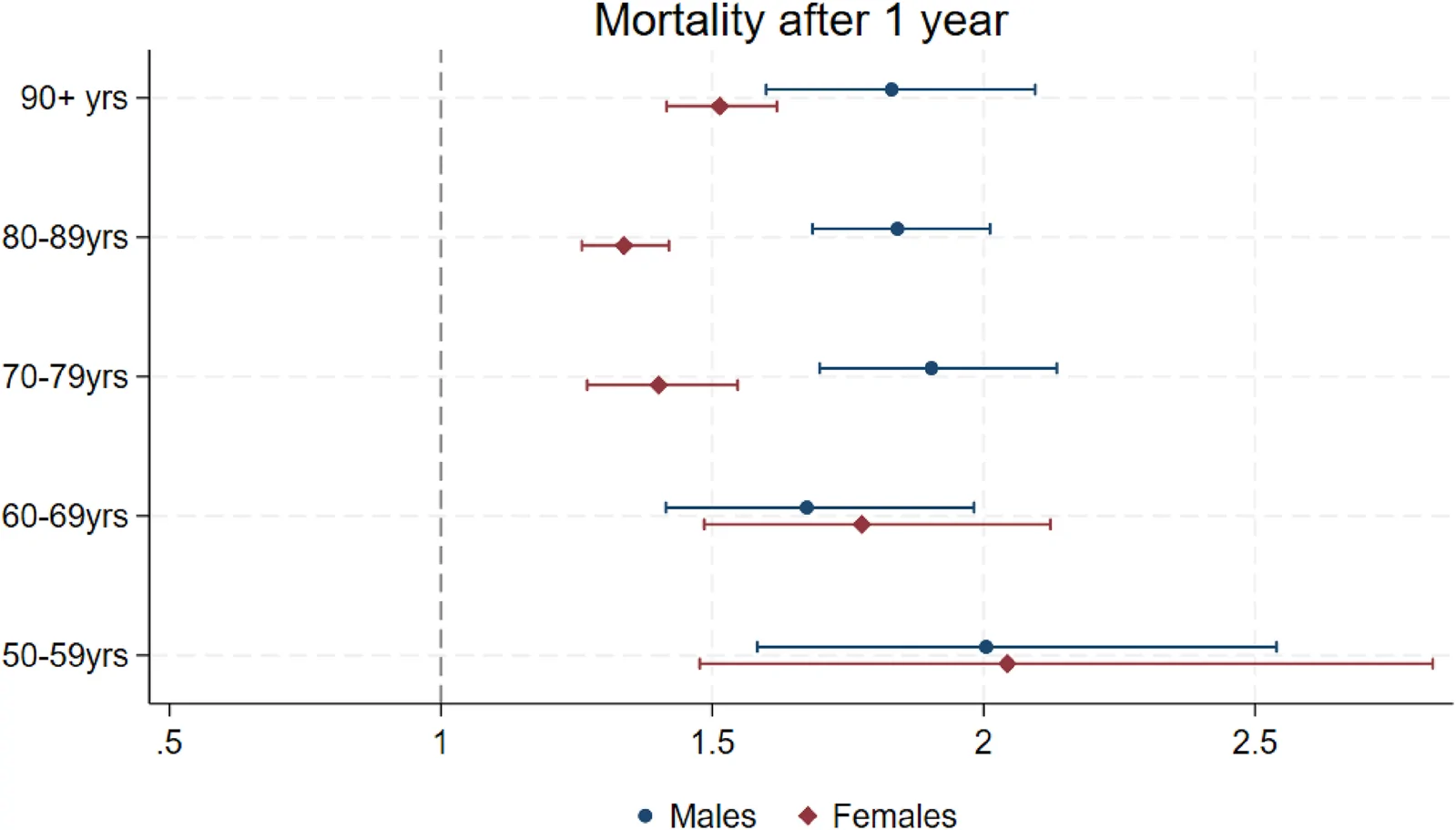
Hazard ratios for death following OVCF stratified by sex, comparing OCVF patients to matched controls. Hazard ratios were estimated using Cox proportional hazards regression and adjusted for age, Charlton Comorbidity categories, region of residence, and education. O Males ◊ Females.

**Fig. 3:**
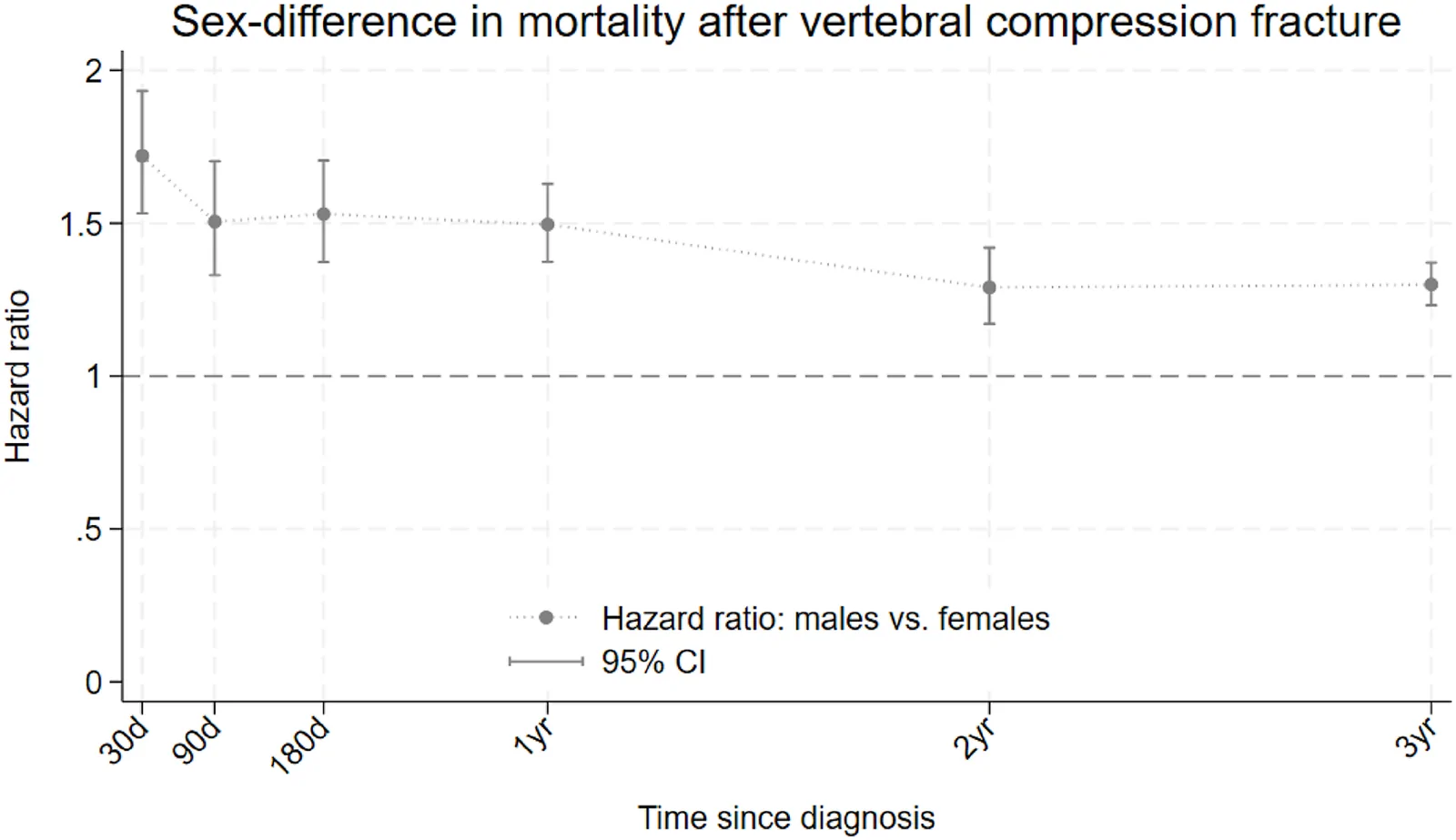
Hazard ratios for death at different times after the index date for males with OVCF compared to females with OVCF estimated with Cox Regression, adjusted for age, Charlton Comorbidity categories, region of residence, and education. Females are the reference group and assigned an hazard ratio of 1.0.

## 4 Discussion

In this nationwide registry-based cohort study, we found that individuals with OVCF had a higher mortality risk than individuals without OVCF of similar sex, age, comorbidity, and education. This was more pronounced in males. Males with OVCF had substantially increased mortality following OVCF compared to females with OVCF. The increased risk persisted even after adjusting for age and comorbidities. These findings indicate a sex difference in post-fracture mortality.

The mortality gap between OVCF patients and controls was most pronounced during the first months after fracture, suggesting a substantial short-term mortality burden associated with OVCF. However, the presence of excess mortality up to 3 years indicate that the adverse impact of OVCF extends well beyond the acute phase.

OVCFs are among the most common fragility fractures and are associated with substantial morbidity and mortality. Multiple large cohort studies have demonstrated that vertebral fractures predict increased all-cause mortality, independent of age and other fracture types (Center et al., 1999; Haentjens et al., 2010; Hasserius et al., 2005). In a study by Center et al., the authors found that vertebral fractures were associated with mortality risks comparable to hip fractures in both sexes (Center et al., 1999). Subsequent research confirmed that this excess risk persists for years after the fracture, regardless of fracture type, and is not fully explained by pre-existing comorbidities (Kado et al., 2003; Bliuc et al., 2009; Gold et al., 2023).

This current study showed similar results as prior studies but more importantly shows higher mortality in men compared to women after an OVCF. Several mechanisms may contribute to the increased mortality found in men versus women. First, men sustaining an OVCF are often more frail at baseline. Second, osteoporosis in men is underdiagnosed and possible undertreated. Men may not receive anti-osteoporotic therapy or relevant fracture-prevention and relevant post fracture rehabilitation, impairing recovery, and increasing vulnerability to complications (Rinonapoli et al., 2021).

Even when adjusting for comorbidities based on the Charlson Comorbidity Index (Charlson et al., 1987, 2022), which in theory provide similar groups for the comparison, a systematic bias may still be present, as men generally engage less with general practitioners and outpatient care. Chronic conditions in men may be under-diagnosed and not reported in the patient registry (Bertakis et al., 2000; Green and Pope, 1999). As a result, men may appear healthier than they truly are, possibly leading to residual confounding, and a potential overestimation of the gender gap in mortality.

As the study is based on the national patient registry (NPR) and the national CPR register, the database only contains data on patients who were diagnosed with vertebral fracture at a hospital acute inpatient or outpatient department. This potentially underestimates the true incidence of osteoporosis in the general population, as osteoporotic fractures are often silent and underdiagnosed. Additionally, fracture severity or functional outcomes were not available, limiting the ability to stratify risk or assess the clinical decision-making processes. As this is a study based on the NPR, we do not have access to objective measures of osteoporosis, however the Danish National guideline stipulates that Osteoporosis may be diagnosed either by a DXA T-score ≤ −2.5 or by the presence of a low-energy hip or vertebral fragility fracture, irrespective of BMD(Andersen, 2025).

A major strength of this study is its nationwide scope, including all patients with clinically diagnosed OVCFs over nearly a decade, ensuring high generalizability within Denmark. The use of national registry data also allows for longitudinal follow-up.

The findings of the current study add to the body of evidence that vertebral fractures independently predict mortality, and that men face a disproportionately higher risk. Clinically, every OVCF should trigger comprehensive fracture liaison evaluation, fall prevention and osteoporosis treatment to mitigate the increased morbidity and mortality risk.

## 5 Conclusion

In conclusion, we found significantly increased mortality of patients with OVCFs compared to matched controls without OVCF. Furthermore, males diagnosed with OVCFs are associated with an almost two-fold higher risk of all-cause mortality three years after diagnosis compared to females. Increased awareness of osteoporosis in the male population and related morbidity should be further addressed in research and clinical practice.

## Ethics declaration

The authors have NOT obtained informed consent from participants or their legal representatives. The current study is a registry-based study, with data available in the Danish National Patient Registry, and thus no informed consent is available or required.

This study was performed in compliance with relevant laws, regulatory frameworks and guidelines where the research took place. Ethics committee approval was not required under relevant laws and institutional guidelines. The study was approved by the Hospital Review Board and the Danish Data Protection Agency (jf.nr 22/41225).

## Funding

Center for Spine Surgery and Research, 10.13039/501100022415Lillebaelt Hospital has received a research grant from Stryker European Operations Limited. No funding has been obtained from: National Institutes of Health (NIH); Wellcome Trust; Howard Hughes Medical Institute (HHMI).

## Declaration of competing interest

The authors declare the following financial interests/personal relationships which may be considered as potential competing interests:Andreas K. Andresen reports was provided by Stryker European Operations Limited. Leah Y. Carreon reports a relationship with Orthopedic Research fund that includes: consulting or advisory. Leah Y. Carreon reports a relationship with Editorial Advisory Board, The Spine Journal that includes: board membership. Institutional grant from Danish Independent Research Foundation. Institutional grant from Cerapedics US. If there are other authors, they declare that they have no known competing financial interests or personal relationships that could have appeared to influence the work reported in this paper.
